# The Clock Genes Are Involved in The Deterioration of Atopic Dermatitis after Day-and-Night Reversed Physical Stress in NC/Nga Mice

**DOI:** 10.2174/1874091X01812010087

**Published:** 2018-06-29

**Authors:** Keiichi Hiramoto, Kumi Orita, Yurika Yamate, Emiko Kasahara, Satoshi Yokoyama, Eisuke F. Sato

**Affiliations:** 1 Department of Pharmaceutical Science, Suzuka University of Medical Science, 3500-3 Minamitamagakicho, Suzuka, Mie 513-8670, Japan; 2 Department of Orthopedic Surgery, Osaka City University Graduate School of Medicine, 1-4-3 Asahimachi, Abeno-ku, Osaka 545-8585, Japan; 3 Department of Pharmaceutical Sciences, Osaka University Graduate School, 1-6 Yamadaoka, Suita, Osaka 565-0871, Japan; 4 Department of Pharmaceutical Sciences, Gifu Pharmaceutical University, 1-25-4 Daigakunishi, Gifu 501-1196, Japan

**Keywords:** Atopic dermatitis, Period 2, Clock, Brain and muscle arnt-like protein 1, Retinoic acid-related orphan receptor α, Day-and-night reversal physical stress

## Abstract

**Background:**

In modern society, irregular lifestyles are a problem. It is well known that Atopic Dermatitis (AD) occurs during physical stress in people with an irregular lifestyle. We evaluated the influence that day-and-night reversal physical stress has on AD.

**Methods:**

Six-week-old specific-pathogen-free and conventional NC/Nga male mice were used. For the day-and-night reversal procedure, the mice ran on a treadmill at a slow speed of 10 m/min for 12 h (between 8:00 and 20:00). Then, between 20:00 and 8:00, we put the mice in a dark place. This treatment was repeated every day for two weeks. The behavioral circadian rhythm of the mice was evaluated with the open field test. Then, the mice were sacrificed and histological examinations of the tissues, the expression of peptide hormones, corticosterone, Immunoglobulin E, histamine, and cytokines was performed using an enzyme-linked immunosorbent assay.

**Results:**

In the treadmill-treated conventional NC/Nga mice, AD symptoms were deteriorated compared with the non-treated conventional NC/Nga mice. The levels of Period (Per) 2, Clock, and brain and muscle arnt-like protein 1 (Bmal1) in the skin were increased constantly in the treadmill-treated conventional mice. Furthermore, the expression of Retinoic Acid-related Orphan Receptor (ROR)α, which activates Bmal1, was increased in the treadmill-treated conventional mice compared with the non-treated conventional mice. In addition, when non-treated conventional mice were administrated by the agonist of RORα, AD symptoms were deteriorated similar to treadmill-treated conventional mice.

**Conclusion:**

In the day-and-night reversal mice, the clock genes were increased constantly, indicating that this is a factor that deteriorated AD.

## 1 INTRODUCTION

Atopic Dermatitis (AD) is a sickness presented as chronic inflammation and itchiness along with dryness of the skin and deviation of barrier function. Many patients have an atopic disposition. In addition, AD and stress are closely related. AD is especially affected by moral and physical stress. If an individual is exposed to moral and physical stress, the Corticotropin-Releasing Hormone (CRH) is secreted in the cerebral hypothalamus [1-3]. Subsequently, the Adrenocorticotropic Hormone (ACTH) is secreted by the pituitary glands and cortisol is secreted by the adrenal glands [4-6]. Furthermore, epidermic cells and fibroblasts also produce cortisol [7]. Cortisol in the skin passively increased due to cortisol from the adrenal glands. In addition, epidermic cells have ACTH receptors, leading to the increase of cortisol in localized skin [8-11]. As a result, the proteins related to skin barriers, such as filaggrin and involucrin are decreased [12]. If the function of the skin barrier fails, the skin can easily receive a stimulus, leading to the spread of AD.

On the other hand, the biological clock is involved in the allergies. In some allergic diseases, there is a time zone in which a symptom deteriorates easily [13, 14]. A modern human being, whose lifestyles include all-night sitting and staying awake late at night, has a different life rhythm than that of people in the past. It is thought that this abnormal biological cycle induces psychological stress. The clock gene is reported to be involved in this circadian rhythm. Periods (Per), Clock, Brain and Muscle Arnt-like protein 1 (Bmal1), and cryptochrome (Cry) exist in the clock genes, and the fundamental mechanism of the circadian rhythm has become clear [15, 16]. Furthermore, a clock gene not only exists in the Suprachiasmatic Nucleus (SCN) of the hypothalamus within the brain, but it also exists in the local organ, and the central clock controls the peripheral clock [17, 18]. The circadian rhythm of the corticosterone concentration in blood disappears with the removal of the SCN, which is the center of this internal clock system in mice. The circadian rhythm of corticosterone is linked to the circadian rhythm of the allergy. Therefore, it was shown that the variation of an allergy is regulated by the internal clock system in the SCN [19].

At the present, although research has progressed on the connection between the clock gene and allergies, few reports have considered the relationship between the clock gene and AD. In this study, we observed mice performing day-and-night reversal treadmill exercise and examined the influence of the clock gene on AD.

## 2 MATERIALS AND METHODS

### 2.1 Animal Experiments

Specific-pathogen-free (SPF) and conventional NC/Nga male mice (6 weeks old) were obtained from SLC (Hamamatsu, Shizuoka, Japan). They were housed in rooms with temperatures of 23 ± 1^o^C; the SPF mice were housed under SPF conditions and the conventional mice were housed under conventional (air-uncontrolled) conditions. Furthermore, these animals were subjected to experiments according to the animal care regulations of Osaka City University Medical School. All procedures were performed with the mice under pentobarbital anesthesia, and all possible efforts were made to minimize suffering. As expected, all of the conventional NC/Nga mice spontaneously started to exhibit symptoms that were characteristic of AD at 7 weeks of age.

The AD symptom score in the conventional mice was determined based on the severity of edema, erythema, and hemorrhage (0, none; 1, slight; 2, moderate; and 3, severe) as described previously [20]. In this study, we divided the mice into four groups (n=6 for each group) and used them at the same time. The four groups are 1. SPF NC/Nga mice (-AD) + non-treadmill, 2. SPF NC/Nga mice (-AD) + treadmill, 3. Conventional NC/Nga mice (+AD) + non-treadmill and 4. Conventional NC/Nga mice (+AD) + treadmill.

### 2.2 Day-and-night Reversal Method

**Fig. (1):**
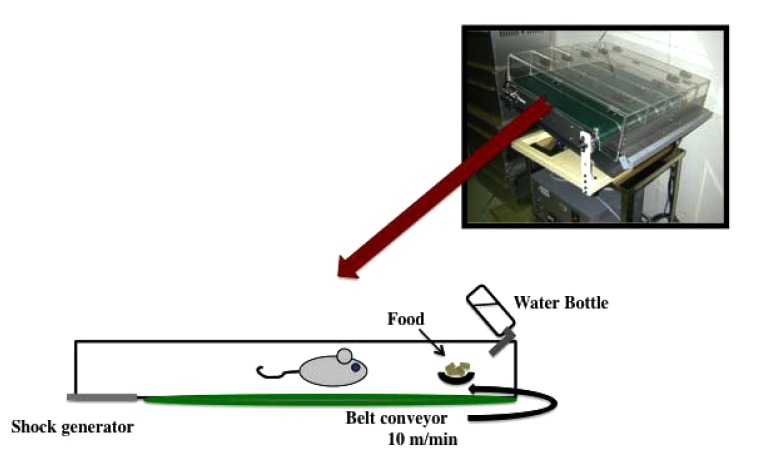
The day-and-night reversal procedure on a treadmill.

Due to the nocturnal nature of the mice, we ran them during the day in order to day-and-night reversal stress. In particular, we run the mice on a treadmill at a slow speed of 10 m/min for 12 h (between 8:00 and 20:00) and put the mice in a dark place to rest for 12 h (between 20:00 and 8:00) (Fig. **1**). This treatment was repeated every day for two weeks. The motor activity of the mice was measured every hour using a video tracking system (Smart2, Bio Research Center, Nagoya, Japan). In addition, there was no difference between treadmill treated group at night and non-treadmill treated group (data not shown).

### 2.3 Open Field Test (OFT)

We performed the OFT in order to investigate the circadian rhythm of the behavior of the mice. The open field area (50 x 50 x 40 cm^3^) was made of plastic. The motor activity of the mice was measured over a 60 min period using a video tracking system (Smart2, Bio Research Center, Nagoya, Japan).

### 2.4 SR1078 Treatment

We used SR1078 (N-[4-(2,2,2-trifluoro-1-hydroxy-1-(trifluolomethyl) ethyl) phenyl]-4-(trifluoromethyl) benzoamide; Tocris, Minneapolis, MN, USA) in order to activate the clock gene. The unclear receptors of retinoic acid-related orphan receptor (ROR)α and RORγ play an integral role in regulating the expression of core clock proteins that drive rhythms in activity and metabolism. SR1078 is a systemic RORα/γ agonist with *in vivo* activity [21]. SR1078 (10 mg/kg/day) was dissolved in dimethyl sulfoxide, which was intraperitoneally injected into treadmill-treated -AD and +AD mice on each day of the experiment [21].

### 2.5 Preparation and Staining of The Skin

For the histological studies, the mice were sacrificed two weeks after the start of the experiment. The skin specimens were fixed in phosphate-buffered paraformaldehyde (4%), embedded in a frozen Tissue-Tek optimal cutting temperature compound (Sakura Finetek, Tokyo, Japan) and cut into 5-μm-thick sections. The sections were stained with hematoxylin & eosin according to the established procedure for the histological analysis of tissue.

Skin specimens were also subjected to immunostaining. We described the details of the staining method in our previous study [22]. Briefly, the specimens were incubated for 2 h at 25^o^C with one of the following primary antibodies: rat monoclonal anti-I-A/I-E (a marker of dendritic cells [DC], 1:100; BD Biosciences, San Jose, CA, USA), goat polyclonal anti-mast cell (MC) tryptase (a marker of MCs, 1:1000; Santa Cruz Biotechnology Inc., Santa Cruz, CA, USA), and rabbit monoclonal anti-GATA3 (a marker of T helper [Th]-2, 1:100; Cell Signaling Technology Inc., Danvers, MA, USA). The specimens were then incubated at 25^o^C for 2 h with fluorescein isothiocyanate-conjugated anti-rat, anti-goat, or anti-rabbit immunoglobulin (1:30; Dako Cytomation, Glostrup, Denmark). The expression levels of I-A/I-E, MC tryptase, and GATA3-positive cells were evaluated immunohistochemically using a fluorescent microscope. The number of I-A/I-E, MC tryptase, and GATA3 was determined using the software program Image J (National Institute of Health, Bethesda, MD, USA).

### 2.6 Western Blotting Analysis

The skin samples were homogenized in a lysis buffer (Kurabo, Osaka, Japan) and centrifuged at 8,000 g for 10 min. We performed a Western blotting analysis as previously described [23]. Briefly, the membranes were incubated at 25^o^C for 1 h with primary antibodies against RORα (1:1,000; GeneTex, Irvine, CA, USA) or β-actin (1:5,000; Sigma-Aldrich, St. Louis, MO, USA). The membranes were then treated with a horseradish peroxidase-conjugated secondary antibody (Novex, Frederick, MD, USA). The immune complexes were detected using an ImmunoStar Zeta reagent (Wako, Osaka, Japan), and images were acquired using the Multi-Gauge software program (Fujifilm, Greenwood, SC, USA).

### 2.7 Analysis of The Levels of Peptide Hormones, Corticosterone, IgE, Histamine, and Cytokines Using an Enzyme-Linked Immunosorbent Assay (ELISA)

Blood samples were obtained from the hearts of the mice two weeks after the treadmill treatment. The plasma levels of ACTH, β-endorphin, α-melanocyte stimulating hormone (α-MSH), corticosterone, IgE, transforming growth factor (TGF)-β, histamine, tumor necrosis factor (TNF)-α, interleukin (IL)-4, IL-10, interferon (IFN)-γ, and IL-13 were determined using commercial ELISA kits (ACTH, β-endorphin and α-MSH: Phoenix Pharmaceuticals Inc., Burlingame, CA, USA; corticosterone: Assaypro, St. Charles, MO, USA; IgE: Yamasa Shoyu Co., Ltd., Chiba, Japan; TGF-β: Promega, Madison, WI, USA; histamine: Bertin Pharma, Montigny le Bretonneux, France; TNF-α, IL-4, IL-10, IFN-γ, and IL-13: R&D Systems, Minneapolis, MN, USA) according to the manufacturers’ instructions. The optical density was measured with a microplate reader (Molecular Devices, Sunny Vale, GA, USA).

### 2.8 RNA Analysis

The RNA assay procedure was based on Li *et al.* and Zheng *et al.* study [24, 25]. Briefly, total RNA was extracted using TRIzol (Invitrogen, Waltham, MA, USA) and TaqMan reverse transcription reagents T (Applied Biosystems, Branchbury, NJ, USA), following the manufacturers’ recommendations. cDNA was then amplified using real-time polymerase chain reaction (RT-PCR) using AmpliTaq Gold DNA Polymerase (Applied Biosystems). For RNA quantification, qRT-PCR amplifications were performed at 95^o^C for 30s, 60^o^C for 30s, and 72^o^C for 30s using specific primers for the housekeeping gene β-action and the three key clock genes: Bmal1, Clock, and Per2. The PCR primers were based on published cDNA sequences (Table **1**). The expression of genes was calculated using the method of Schmittgen *et al.* [26].

### 2.9 Statistical Analyses

All data are presented as the mean ± standard deviation. The results were statistically analyzed using Microsoft Excel 2010 and a one-way analysis of variance followed by Tukey’s post hoc test using the software program SPSS, version 20. Results were considered significant at p-values of <0.05.

## 3 RESULTS

### 3.1 Influence of Treadmill Exercises on The Circadian Rhythm of NC/Nga Mice

**Fig. (2A):**
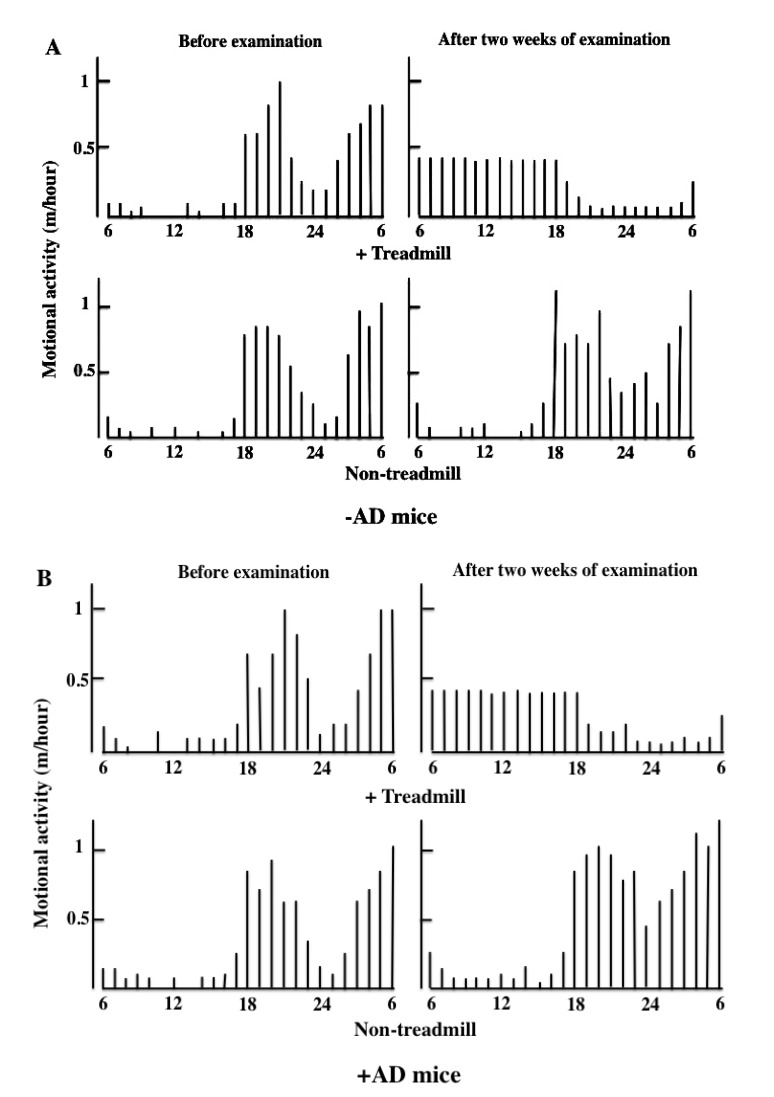
Influence of the treadmill treatment (day-and-night reversal stress) on the circadian rhythm of motional activety in –AD and +AD mice. The mice ran on a treadmill for 12 h during the daytime (8:00 am-8:00 pm). These treadmill treatments were performed for two weeks. The data show typical experiments of a treadmill and non-treadmill mouse. **(A)** –AD mouse (SPF NC/Nga mice), **(B)** +AD mouse (conventional NC/Nga mouse).

During the daytime, mice were not put to sleep, and we measured motor activity for 12 h every time the treadmill was used until bedtime. Two weeks after the treadmill training, the motor activity at night was decreased in the day-and-night reversal of the -AD and +AD mice (Fig. **2**).

### 3.2 Evaluation of The Severity of AD in Treadmill-Treated Mice

**Fig. (3):**
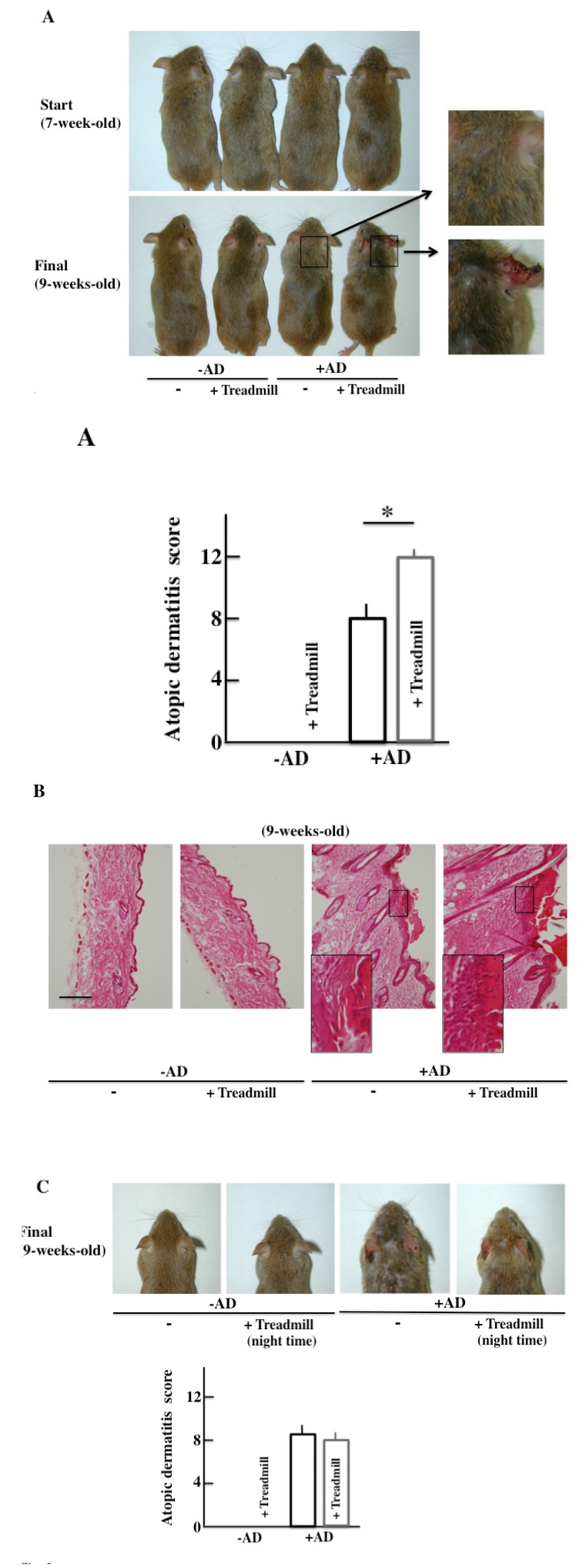
Effect of day-and-night reversal stress on skin symptoms and AD scores, and histological analysis of the skin. **A)** Skin symptoms and AD scores after the two-week treadmill treatment. **B)** A histological analysis of skin tissue actions after the treadmill treatment. **C)** The mice ran on a treadmill for 12 h at nighttime (8:00 pm-8:00 am). Skin symptoms and AD scores after the two-week treadmill treatment. –AD: SPF NC/Nga mice, +AD: conventional NC/Nga mouse, AD: Atopic dermatitis. The values are presented at the mean ± standard deviations, derived from 6 animals.*, P<0.05. Scale bar = 100 μm.

The -AD mice did not show AD-like symptoms of the skin two weeks after the start of the examination. In the +AD mice, AD-like symptoms were seen after two weeks. Furthermore, the AD-like symptoms deteriorated in the day-and-night reversal NC/Nga mice (Fig. **3A**).

The morphological findings are shown in Fig. (**3B**). Alteration due to day-and-night reversal was not observed in -AD mice. In the +AD mice, notable acanthosis, hyperplasia of the epidermis, ulceration, and infiltration of large numbers of lymphocytes in the dermis were observed. The day-and-night reversal NC/Nga mice had stronger systemic change than the non-treated NC/Nga mice.

The atopic score of +AD mice that had performed treadmill training at night (the activity time of the mice) was not different from that of the non-treated, +AD mice (Fig. **3C**). From this result, it was shown that the physical stress of mice caused by treadmill treatment during daytime deteriorates AD symptoms.

### 3.3 Effect of Day-and-Night Reversal Stress on the AD Symptoms in NC/Nga Mice

**Fig. (4):**
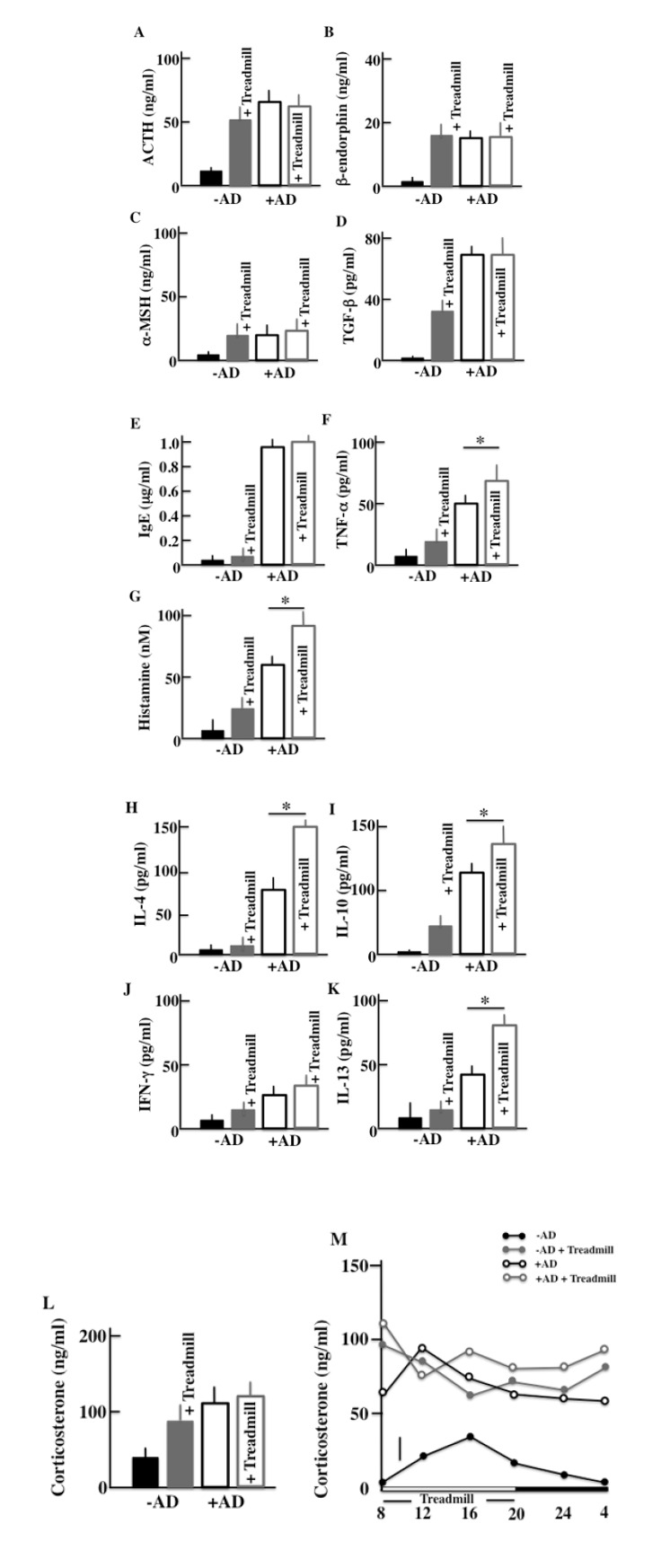
An analysis of the plasma ACTH **(A)**, β-endorphin **(B)**, α-MSH **(C)**, TGF-β (D), IgE **(E)**, TNF-α **(F)**, histamine **(G)**, IL-4 (H), IL-10 (I), IFN-γ (J), IL-13 **(K)**, and corticosterone **(L)** concentrations after the two-week treadmill treatment. In addition, the plasma corticosterone levels were used to investigate the circadian rhythm after the treadmill treatment **(M)**. –AD: SPF NC/Nga mice, +AD: conventional NC/Nga mouse. The values are presented as the mean ± standard deviation, derived from 6 animals. *, P<0.05.

We performed ELISA assays to measure the plasma levels of ACTH, β-endorphin, α-MSH, corticosterone, TGF-β, IgE, TNF-α, INF-γ, histamine, IL-4, IL-10, and IL-13 in the treadmill-treated mice after two weeks of treatment. As for markers of a stress and AD symptoms, we have measured following levels. More specifically, we measured the levels of ACTH, α-MSH, β-endorphin, corticosterone and TGF-β as famous stress hormones secreted from the hypothalamic-pituitary-adrenal axis (HPA axis). And since AD was closely concerned with the mast cell, then we measured the levels of IgE, TNF-α and histamine as cytokine secreted from mast cell. Furthermore, we measured the cytokine secreted from helper T (Th)1 cell (IFN-γ and IL-13) and Th2 cell (IL-4 and IL-10) which is concerned in AD. In the -AD mice, the protein levels in the blood were higher in treadmill-treated mice than in non-treadmill mice. In the +AD mice, the levels of ACTH (Fig. **4A**), β-endorphin (Fig. **4b**), α-MSH (Fig. **4C**), and corticosterone (Fig. **4I**) did not change between treadmill and non-treadmill mice. These levels were not changed between -AD-treadmill mice and non-treadmill +AD mice. In the +AD mice, the levels of IgE (Fig. **4D**), TGF-β (Fig. **4E**), and IFN-γ (Fig. **4J**) did not change between the treadmill and non-treadmill; however, the same levels were higher in +AD mice than in -AD-treadmill mice. On the other hand, in the +AD mice under treadmill, the levels of TNF-α (Fig. **4F**), histamine (Fig. **4G**), IL-4 (Fig. **4B**), IL-10 (Fig. **4I**), and IL-13 (Fig. **4K**) were higher than those in non-treadmill mice. We measured the circadian rhythms of corticosterone in the four groups (Fig. **4M**). Although the plasma corticosterone levels of the treadmill-treated +AD, treadmill-treated -AD, and non-treated +AD mice were higher than those of non-treated SPF mice, a difference among the high level three groups was not observed.

### 3.4 Effects of the Treadmill Training on the Expression of Bmal1, Clock, and Per2 in NC/Nga Mice

**Fig. (5):**
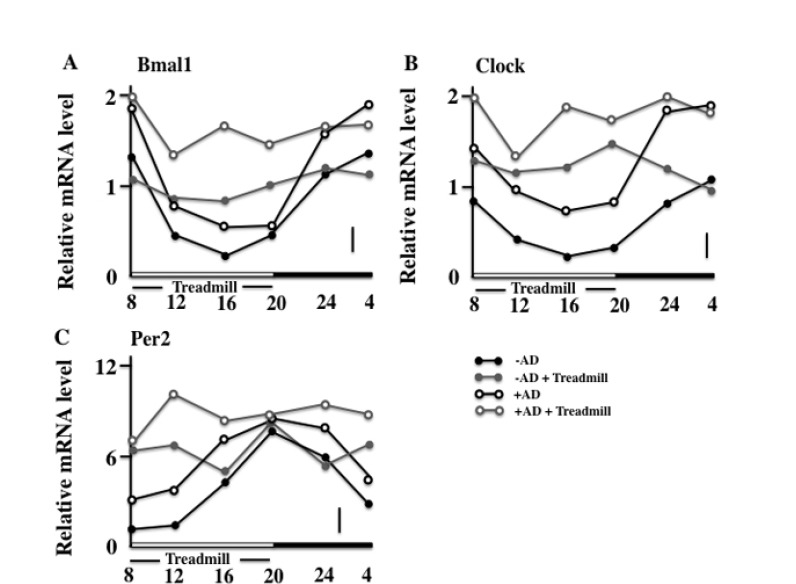
The clock gene RNA follows circadian rhythms in the dorsal skin of mice. The circadian rhythm of the expression of Bmal1 RNA **(A)**, Clock RNA **(B)**, and Per2 RNA **(C)** was no longer observed after the two-week treadmill treatment. –AD: SPF NC/Nga mice, +AD: conventional NC/Nga mouse. The values are presented as the mean ± standard deviation, derived from 6 animals. The bar shows the pooled value and standard error of the mean.

Next, we observed the circadian rhythm of skin expression of the clock genes on the final day of the examination. In the non-treadmill mice, the Bmal1 and Clock were low in the daytime and became high at night; however, in the treadmill-trained mice, a difference between the levels of day and night was not seen. In addition, the +AD treadmill mice maintained high values compared with other groups (Figs. **5A** and **5B**). On the other hand, the expression of the Per2 of both non-treadmill treatment groups had a circadian rhythm (although it disagreed with Bmal1 and Clock). However, in the treadmill treatment, the circadian rhythm of the expression of Per2 of both groups disappeared. In addition, the treadmill-trained +AD mice maintained high values (Fig. **5c**). Furthermore, the cryptochrome (Cry) synchronized with Per2 and had the same rhythm in each group (data not shown).

### 3.5 Effect of the Treadmill Treatment on the Expression of DC, MC, and GATA3 in NC/Nga Mice

**Fig. (6):**
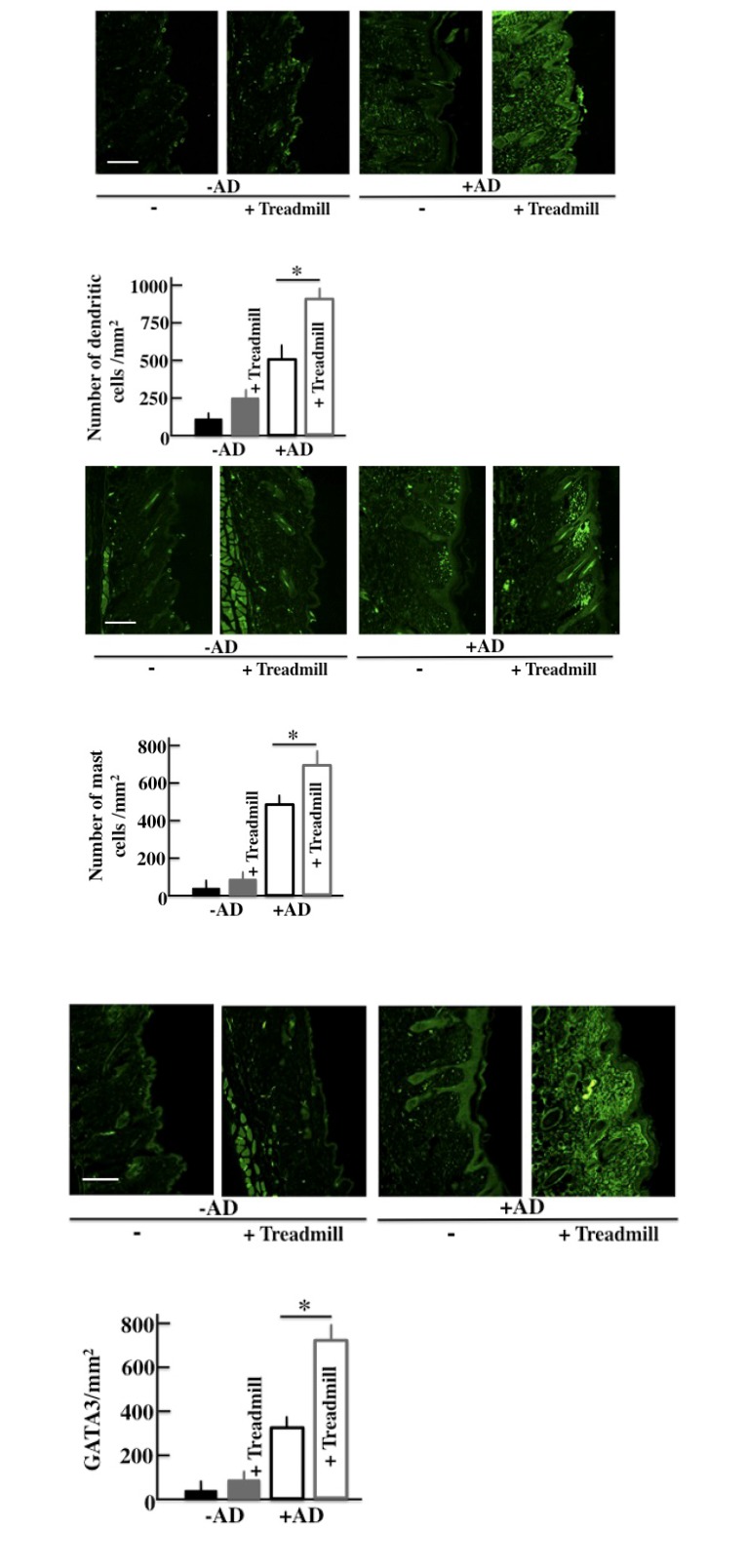
The effect of day-and-night reversal stress on the expression of Dendritic Cells (DCs), Mast Cells (MCs), and GATA3 in the skin. An immunohistochemical analysis of DCs, MCs, and GATA3 and stained DC, MC, and GATA3 counts in the skin after the treadmill treatment for two weeks. –AD: SPF NC/Nga mice, +AD: conventional NC/Nga mouse. The values are presented as the mean ± standard deviation, derived from 6 animals.*, P<0.05. Scale bar = 100 μm.

We investigated the immunohistochemical expression of DC, MC, and GATA3 (a marker of the Th2 cell) [27] in the skin. The expressions of DC, MC, and GATA3 were higher in +AD mice than in -AD mice and were the highest in treadmill-treated +AD mice (Figs. **6A**, **6B** and **6C**).

### 3.6 Effect of The Treadmill Treatment on The Expression of RORα in NC/Nga Mice

**Fig. (7):**
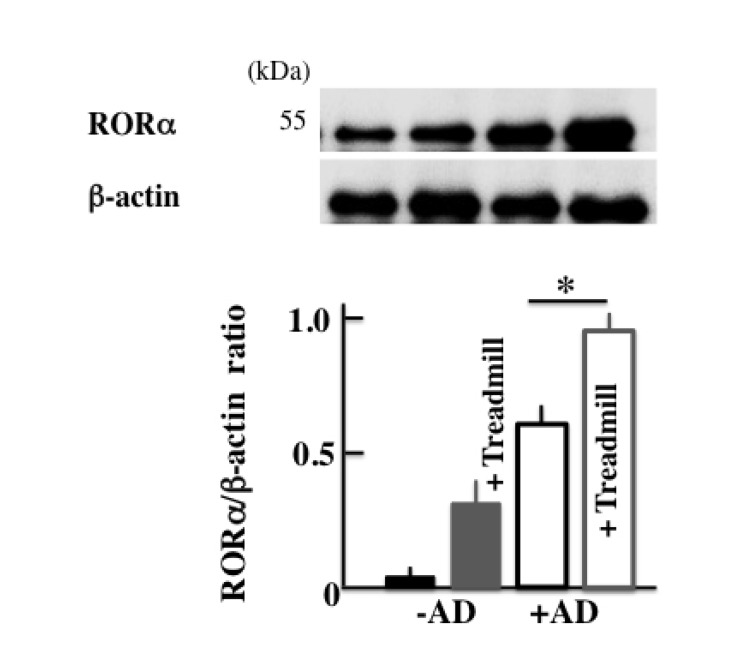
The effects of day-and-night reversal stress on the expression of RORα in the skin. RORα was analyzed using the Western blotting method. –AD: SPF NC/Nga mice, +AD: conventional NC/Nga mouse. The values are presented as the mean ± standard deviation, derived from 6 animals.*, P<0.05.

In order to investigate the cause of the increase in the expression of Bmal1, we investigated RORα, which is a nuclear receptor. The expression of RORα was higher in +AD mice than in -AD mice and was the highest in treadmill-treated +AD mice (Fig. **7**).

### 3.7 AD Symptoms in NC/Nga Mice after SR1078 (Agonist of the RORα/γ) Treatment

**Fig. (8):**
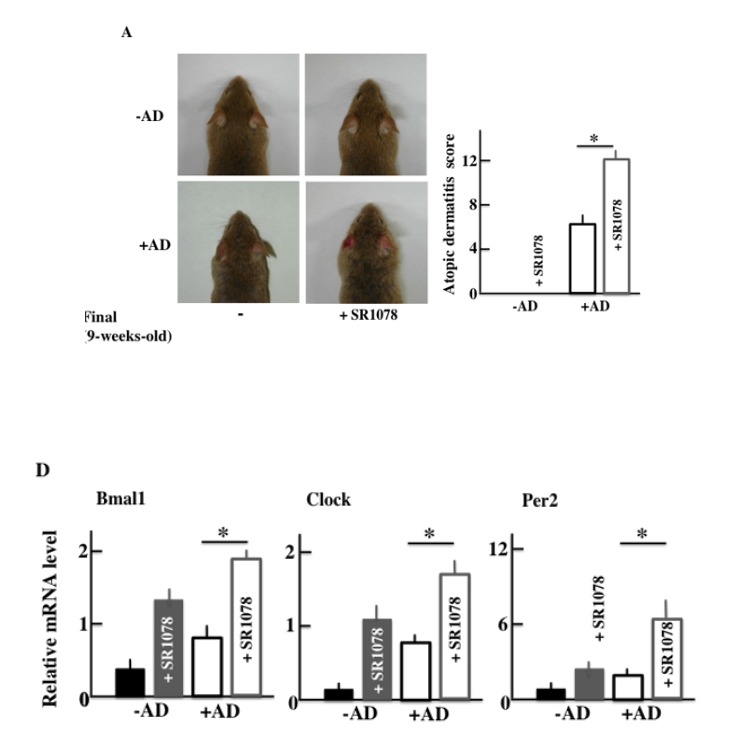
The effects of SR1078 on NC/Nga conventional mice. **(A)** Macroscopic view and atopic dermatitis scores, **(B)** plasma IL-4, **(C)** plasma histamine, and **(D)** the RNA expression of Bmal1, Clock, and Per2 in the skin. –AD: SPF NC/Nga mice, +AD: conventional NC/Nga mouse. The values are presented as the mean ± standard deviation, derived from 6 animals.*, P<0.05.

Furthermore, in order to investigate the effect of AD of the clock genes, we medicated both +AD and -AD mice with an agonist of the RORα/γ, which is one of the clock gene receptors. The +AD mice had higher AD scores than -AD mice (Fig. **8A**), as well as higher levels of plasma in IL-4 and histamine without SR1078 treatment (Figs. **8B** and **8C**). In addition, each value was increased due to the SR1078 treatment. However, the AD score of the -AD mice was not changed. Furthermore, we observed the expression of RNA of the clock genes when SR1078 was injected. The clock genes (Bmal1, Clock, and Per2) were increased due to SR1078 treatment. In addition, the clock genes were the highest in +AD mice that were injected with SR1078 (Fig. **8D**).

## 4 DISCUSSION

In this study, we considered the relationship between clock genes and AD. We performed compulsory day-and-night reversed treadmill treatment in NC/Nga mice (the AD model). In the treadmill-treated +AD mice, AD-like symptoms deteriorated compared with those of untreated +AD mice. In addition, in treadmill-treated +AD mice, the increases of Bmal1, Clock, and Per2 were observed compared with untreated +AD mice. Furthermore, an increase in the expression of RORα was observed in treated mice.

In this study, we thought that chronic stress due to long-term treadmill treatment deteriorated the symptoms of AD. We measured the level of α-MSH, which is a chronic stress marker [28]. As a result, in the +AD mice, we did not observe a difference among non-treadmill, non-revered treadmill, and revered treadmill (Sup. Fig. **1**). Furthermore, on the TGF-β which is a typical marker of chronic stress similarly [29], the difference was not seen between non-treadmill, non-reversed treadmill, and reversed treadmill (data not shown). Therefore, in this experiment, we thought that chronic stress did not influence the deterioration of AD symptoms.

Allergies and the clock genes are connected. The circadian rhythm dictates that an allergy will be most severe at 10:00 am and least severe at 10:00 pm. The circadian rhythm of allergies synchronizes with that of the clock genes [30]. The association between the periodicity of an allergy and the circadian rhythm of an adrenal-gland origin secretor factor (corticosterone) has been reported. For example, the circadian rhythm of corticosterone was lost in the Per2 knockout mice in one study [31]. On the contrary, in this study, the opposition of circadian rhythm of corticosterone occurred due to day-and-night reversal (Figs. **4I** and **M**). On the other hand, the relative amount of corticosterone did not change between non-treadmill treated +AD mice and treadmill-treated +AD mice, but the AD symptoms were remarkably different between these mice (Fig. **3**). Therefore, corticosterone was considered to be indirectly related to the exacerbation of AD. In addition, in this study, we examined the relationship between AD and the clock genes, and one of the parameters was histamine that was secreted from MCs and DCs. However, regarding the histamine H2 and H2 receptor antagonist, there was almost no amelioration of the AD symptoms [32]. Therefore, to determine the factors that lead to degranulation of MCs, we think that it is better to measure leukotriene B4 [33] and tryptase [34], which are closely concerned in the symptom of AD, rather the histamine.

The clock genes appear in all the cellulase containing an immunocyte [30]. In this study, the MC and DC that were involved in the allergy increased in the treadmill-treated +AD mice (Fig. **6**). MCs especially secrete histamine and TNF-α, and they are closely involved in the formation of AD. In wild-type mice, type I allergy has a circadian rhythm, and it is the most severe at 10:00 am and the least severe at 10:00 pm [31]. The circadian rhythm of this type I allergy synchronizes with that of Bmal1 and Clock, and when the expression of Bmal1 and Clock is high, type 1 allergy becomes severe [30, 31, 35]. In our data, in the day-and-night reversal conventional mice, the expression of Bmal1 and Clock was always high (Fig. **5**) and may have always stimulated degranulation of the MCs. Furthermore, E-Box, which is a Bmal1/Clock receptor, combines theoretically in the promoter region of FcεR1β, which is mostly an IgE receptor [35]. If the Clock is knocked down, the expression of FcεR1β and FceR1 will fail and degranulation depression will occur [36]. Based on this, we thought that excessive expression of Bmal1 and Clock activated the MCs and led to an exacerbation of AD.

In addition, an increase in the expression of DCs was observed in this experiment (Fig. **6**). E4BP4, which is a transcription factor of the clock gene, is reported to activate the differentiation of DCs [37]. NC/Nga mice develop the symptoms of AD in conventional conditions. Therefore, the concentration of IL-4 and IL-10 inside the body is high. The high concentration of IL-4 and IL-10 induces Th2 cells selectively, leading to mature DCs [38, 39] (Fig. **6C**). Th2 cells further secrete IL-4, IL-10, and IL-13. These cytokines might activate MC and promote degranulation; and as a result, AD may be exacerbated.

Next, we injected the untreated +AD mice with an agonist of RORα in order to investigate the factors leading to the increase in the expression of Bmal1. RORα activates Bmal1 [40]. In the current study, the expression of RORα was increased in treadmill-treated +AD mice (Fig. **7**). Surprisingly, the deterioration of AD was shown when non-treadmill +AD mice were injected with the RORα agonist (Figs. **8A**, **B** and **C**). The result means that even if non-treadmill +AD mice do not have a physical stress of a day-and-night reversal, it indicates that an atopic deteriorates by the injection of RORα/γ agonist (SR1078). In addition, Bmal1 increased in these mice (Fig. **8D**). These indicated that the increase in RORα possibly caused the deterioration of the symptoms of AD. However, the clock genes are intricately and mutually related, and it is impossible for Bmal1 alone to indicate symptoms. Therefore, it is necessary to investigate the action of each clock genes and their interaction with each other in more detail. In addition, the signal of the increase in RORα regarding AD deterioration is not clear; therefore, we should investigate many more cases.

Based on the above results, we determined that the clock genes activated MCs directly and induced granulation; furthermore, they activated differentiation of DCs. +AD mice had a high level of IL-4. DCs, which specialized and matured in this living body situation, induced Th2 cells selectively. The induced Th2 cells secreted IL-4 further, activating the MCs. In addition, the expression of the nuclear receptor RORα increased, and RORα activated the signal transmission of the clock genes. Thus, we thought that the clock genes and RORα, IL-4, DC, and MC are correlated.

## CONCLUSION

We showed that AD deteriorates due to the physical stress of day-and-night reversal treadmill exercises. During this day-and-night reversal of physical stress, the internal clock was disordered, and the expression of the clock genes (Bmal1, Clock, and Per2) increased. These results indicated that the clock genes were closely related to the onset of AD. In modern society, the expression of clock genes is disrupted due to irregular lifestyles, and AD becomes worse as a result. However, the relation between the clock genes and AD has not been revealed in many cases, and further examination is necessary.

## Figures and Tables

**Fig. (Sp1):**
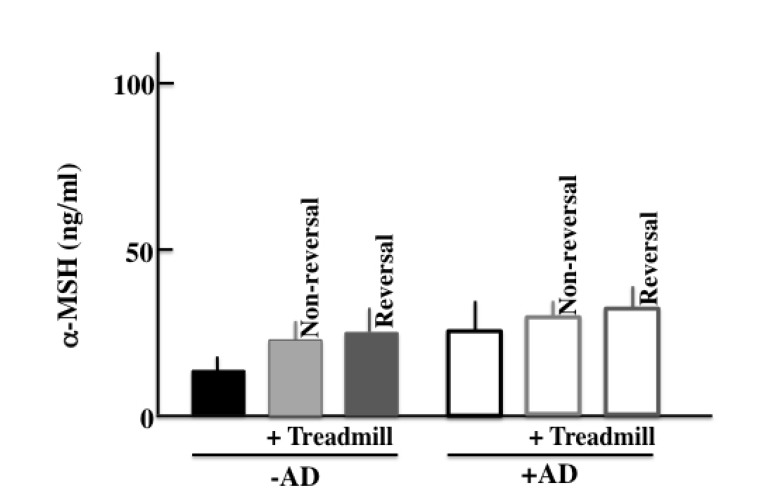
An analysis of the plasma a-MSH concentration after the two-week treadmill treatment. –AD: SPF NC/Nga mice, +AD: conventional NC/Nga mouse. The values are presented as the mean ± standard deviation, derived from 6 animals.*, P<0.05.

**Table 1:** Primer sequences for the real-time polymerase chain reaction.

Gene Gene Bank Forward Reverse
Number
β-actin NM_031144 TTGCCCTAGACTTCGAGCAA CAGGAAGGAAGGCTGGAAGA
Bmal1 NM_024362 TGAACCAGACAATGAGGGCT TATGCCAAAATAGCCGTCGCC
Clock NM_021859 CTCCCCACAAGACTGCAGTA CCTGTGTGGCCTTTACCCTA
Per2 NM_031678 GTCCCCGGCTAGAAGTCTAC TAAACCTCCCCACAGCTCTG
